# Fast principal component analysis for cryo-electron microscopy images

**DOI:** 10.1017/S2633903X23000028

**Published:** 2023-02-03

**Authors:** Nicholas F. Marshall, Oscar Mickelin, Yunpeng Shi, Amit Singer

**Affiliations:** 1 Department of Mathematics, Oregon State University, Corvallis, Oregon 97331, USA; 2 Program in Applied and Computational Mathematics, Princeton University, Princeton, New Jersey 08544, USA; 3 Department of Mathematics, Princeton University, Princeton, New Jersey 08544, USA

**Keywords:** Covariance estimation, cryo-EM, denoising, Fourier–Bessel, principal component analysis, single particle reconstruction

## Abstract

Principal component analysis (PCA) plays an important role in the analysis of cryo-electron microscopy (cryo-EM) images for various tasks such as classification, denoising, compression, and ab initio modeling. We introduce a fast method for estimating a compressed representation of the 2-D covariance matrix of noisy cryo-EM projection images affected by radial point spread functions that enables fast PCA computation. Our method is based on a new algorithm for expanding images in the Fourier–Bessel basis (the harmonics on the disk), which provides a convenient way to handle the effect of the contrast transfer functions. For *N* images of size *L* × *L*, our method has time complexity *O*(*NL*^3^ + *L*^4^) and space complexity *O*(*NL*^2^ + *L*^3^). In contrast to previous work, these complexities are independent of the number of different contrast transfer functions of the images. We demonstrate our approach on synthetic and experimental data and show acceleration by factors of up to two orders of magnitude.

## 1. Introduction

We study the problem of computing a compressed representation of the covariance matrix of 2-D cryo-electron microscopy (cryo-EM) images for the purpose of performing principal component analysis (PCA). More precisely, we consider an image formation model where the measurement *g*_*i*_ is defined by

(1) $$g_{i}=h_{i}*f_{i}+\varepsilon_{i}\;\text{for}\;i=1,\ldots ,N,$$

where *h*_*i*_ is a radial function, * denotes convolution, *f*_*i*_ is a ground-truth image, *ε*_*i*_ is the noise term, and *N* the total number of images. We emphasize that we assume *h*_*i*_ is radial, see Assumption A1.

We are motivated by single particle cryo-EM imaging, which is an important technique for determining the 3-D structure of macromolecules. In particular, the single particle reconstruction (SPR) problem asks to recover the 3-D structure of a macromolecule from noisy 2-D images of its tomographic projections along unknown viewing angles. In cryo-EM, the mathematical model is a special case of (1) and is of the form

(2) $$g_{i}\left(x^{\prime }\right)=h_{i}*\int_{\mathbb{R}}\varphi_{i}\left(R_{i}^{-1}x\right)dx_{3}+\varepsilon_{i}\left(x^{\prime }\right),\;i=1,\ldots ,N,$$

where $x=\left(x^{\prime },x_{3}\right)\in {\mathbb{R}}^{2}\times \mathbb{R}≅{\mathbb{R}}^{3}$ are 3-D spatial coordinates with *x*_3_ representing the projection direction, *h*_*i*_ is the point spread function, $\varphi_{i}:{\mathbb{R}}^{3}\to \mathbb{R}$ is the electrostatic potential of a molecule, *R*_*i*_ ∈ *SO*(3) is a 3-D rotation, and *ε*_*i*_ the noise term. In computational microscopy, it is typical to work with the Fourier transform of the point spread function, which is known as the contrast transfer function (CTF). In the simplest case, each measurement could correspond to a single fixed molecule potential function *φ*_*i*_ = *φ*; however, in general, we may assume that each *φ*_*i*_ could be a random variable representing a mixture of molecules, conformational heterogeneity, cases where the images are not perfectly centered, or other measurement imperfections^(1,2)^.

In general, each measurement *g*_*i*_ can be associated with a different point spread function; however, in practice, a group of measurements, called a defocus group, can share a common point spread function. We assume that the measurements are grouped into *M* ≤ *N* defocus groups. Given *g*_*i*_ and *h*_*i*_ for *i* = 1, …, *N*, our goal is to estimate the 2-D covariance function $c:{\mathbb{R}}^{2}\times {\mathbb{R}}^{2}\to \mathbb{R}$ of the images

(3) $$c\left(x^{\prime },y^{\prime }\right)≔E\left[\left(f\left(x^{\prime }\right)-\bar{f}\left(x^{\prime }\right)\right)\left(f\left(y^{\prime }\right)-\bar{f}\left(y^{\prime }\right)\right)\right],$$

where *f* is a random variable from the same distribution as the images *f*_*i*_, and $\bar{f}\left(x^{\prime }\right)=E\left[f\left(x^{\prime }\right)\right]$. We assume that the distribution of the images is invariant to in-plane rotations (which is typically the case in cryo-EM).

In cryo-EM, the random variable *f* is of the form $f\left(x^{\prime }\right)=\int_{\mathbb{R}}\Phi \left(R^{-1}x\right)dx_{3}$, where the random variable *R* is an unknown viewing angle, and the random variable *Φ* is a molecule potential. There is generally no physical reason for a molecule to prefer one in-plane rotation to another so distributions of random variables of this form are generally invariant to in-plane rotations. In the field of cryo-EM processing, the covariance function *c* is simply referred to as the 2-D covariance.

### 1.1 Motivation

The 2D-covariance is an essential component of a number of computational techniques in cryo-EM; we survey a few of these below.

First, we are motivated by PCA, which is a ubiquitous technique in statistics, data science, and computational mathematics and has applications to dimensionality reduction, denoising, visualization, among others. The principal components (i.e., the top eigenvectors of the digitized covariance matrix) have a number of uses in the computational cryo-EM pipeline. The subspace corresponding to the top eigenvectors of the covariance matrix identifies salient features of the dataset which enables, for instance, improved methods for image classification and visualization, such as Multivariate Statistical Analysis^(3–6)^. These techniques improve computational speed, since clustering becomes computationally easier in a space of reduced dimension, as well as accuracy, since dimensionality reduction by PCA amplifies the effective signal-to-noise ratio (SNR) because many coordinates for which noise dominates the signal are eliminated^(7)^.

Second, the covariance matrix has applications in the method of moments, a classical statistical inference method, applied to cryo-EM^(8)^. In this method, the 2-D covariance is used to compute the similarly defined autocorrelation function of the underlying 3-D structure. Under further assumptions such as sufficient nonuniformity of the distribution of the viewing angles^(9)^ or sufficient sparsity of the molecular structure^(10)^, this autocorrelation function determines the 3-D density map either up to a finite list of possible structures or uniquely, respectively. This has been further developed into principled methods for ab initio estimation of cryo-EM structures^(9,10)^, with reduced risk of user-induced model bias in the initial model. Alternatively, when additional information is available, for instance, one^(11)^ or two^(12)^ noiseless projection images, or the 3-D structure of a related, homologous structure^(13,14)^, the 3-D density map is uniquely determined by the autocorrelation, without requiring any structural assumptions.

Third, the covariance matrix has applications in denoising and CTF-correcting projection images. Covariance Wiener Filtering (CWF)^(15)^ is an approach that uses the classic Wiener filtering framework with the estimated covariance matrix to solve the image deconvolution and denoising problem. The technique represents images in a lower dimensional subspace that is formed from PCA using the estimated covariance matrix. The method then applies Wiener filtering to correct the CTFs and denoise the images in this reduced subspace.

Compared to the standard PCA problem, the cryo-EM setting exhibits further computational challenges, since the estimation method also has to account for convolution with the point spread function, which destroys information of the resulting convolved function; see Section Section 2.2 for a more precise statement. On the other hand, the problem has additional symmetries making fast algorithms possible. In this paper, we present a new fast algorithm for estimating the covariance matrix that improves upon past approaches (especially when there are a large number of defocus groups) in terms of time and space complexity.

### 1.2. Main contribution

The main contribution of this paper is a new computational method for estimating the covariance Equation (3) from *N* measurements of the form Equation (1) encoded by *L* × *L* digitized images. The presented fast method has time complexity *O*(*NL*^3^ + *L*^4^) independent of the number *M* ≤ *N* of defocus groups. This is in contrast to past methods, where this complexity scales poorly with *M* and involves *O*(*MTL*^4^ + *NL*^3^) operations^(15)^, where *T* is the number of iterations needed in a Conjugate Gradient step. Many modern cryo-EM experimental datasets fall into the computationally challenging regime where *M* scales with *N*.

Our fast method hinges on a new fast and accurate method for expanding *L* × *L* images into the Fourier–Bessel basis, which provides a convenient way to handle convolution of radial functions (such as point spread functions) with images: namely, convolution with radial functions can be expanded as a diagonal operator operating on the basis coefficients^(16)^.

The Fourier–Bessel basis functions are *harmonics* on the disk: the standing waves associated with the resonant frequencies of a disk-shaped drum with a fixed boundary. More precisely, the harmonics on the disk are eigenfunctions of the Laplacian on the unit disk that satisfy Dirichlet boundary conditions. In computational mathematics, this basis is referred to as the Fourier–Bessel basis, since the basis functions can be expressed as a product of a Bessel function and a complex exponential; see Equation (6) for a definition.

Because of this simple structure, the covariance matrix of clean images can be estimated by a simple closed-form solution, without using the (computationally expensive) conjugate gradient method from previous approaches. Simultaneously, the covariance matrix retains its block diagonal structure, meaning that its diagonal blocks can be estimated separately and independently, which altogether makes PCA fast.

We present numerical results of covariance estimation on synthetic and experimental data. Additionally, we show how the estimated covariance matrix can be used to denoise images using CWF, and perform PCA to visualize eigenimages from experimental data. Code implementing the method is publicly available online.^1^ Moreover, our approach has the potential to generalize to settings beyond cryo-EM, where PCA is used for signals estimated under more general group actions^(17)^.

The remainder of the article is organized as follows. In Section 2, we describe the computational method. In Section 3, we present numerical results for synthetic data. In Section 4 we present numerical results for experimental data. In Section 5, we discuss the results and possible extensions.

## 2. Methodology

### 2.1. Notation

For two *M* × *N*-matrices *A* and *B*, we denote their Hadamard (or entrywise) product by *A* ⊙ *B*, the Hadamard division of *A* and *B* by *A* ⊘ *B* and the *ℓ*th Hadamard power of *A* by A^⊙*ℓ*^. These operations are defined elementwise by

(4) $${\left(A⊙B\right)}_{jk}=A_{jk}B_{jk},\;{\left(A⊘B\right)}_{jk}=\frac{A_{jk}}{B_{jk}},\;{\left(A^{⊙\ell }\right)}_{jk}=A_{jk}^{\ell },$$

respectively. If *w* is an *N*-dimensional vector, then diag(*w*) denotes the *N* × *N* matrix with w along its diagonal, that is, diagonal, that is, diag(*w*)_*jj*_ = *w*_*j*_, and zeros elsewhere. If $f:{\mathbb{R}}^{2}\to \mathbb{R}$ is a radial function, we write *f*(*x*) = *f*(|*x*|) to mean that *f* can be expressed as a function only of the magnitude |*x*| of *x*.

For an integrable function $f:{\mathbb{R}}^{2}\to \mathbb{R}$, we denote the Fourier transform of *f* by $\hat{f}:{\mathbb{R}}^{2}\to \mathbb{C}$ with the convention that

(5) $$\hat{f}(\xi )=\frac{1}{2\pi }\int_{{\mathbb{R}}^{2}}f(x)e^{-ix\cdot \xi }dx.$$

### 2.2 Technical details

We make the following assumptions

A1. We assume that the point spread functions *h*_*i*_ in the model Equation (1) are radial functions; this implies that their Fourier transform (the CTFs) are also radial. In systems where astigmatism is present and the point spread function deviates slightly from a radial function, our approach can be used as an initial approximation that could be refined using the Conjugate Gradient method.
A2. We assume that the underlying images *f*_*i*_ in the model Equation (1) are i.i.d. random variables whose distribution is invariant to in-plane rotations.
A3. We assume a technical condition on the Fourier transform of the point spread functions *h*_*i*_ in the model Equation (1). Namely, that the Fourier transforms ${\hat{h}}_{i}$ of the *h*_*i*_ satisfy

$$\underset{\left|\xi \right|,\left|\eta \right|\in \left[\lambda_{\text{min}},\lambda_{\text{max}}\right]}{\text{inf}}\sum_{i=1}^{N}{\left|{\hat{h}}_{i}(\xi )\right|}^{2}{\left|{\hat{h}}_{i}(\eta )\right|}^{2}\geq \delta ,$$

where *δ* > 0 is fixed and [*λ*_min_, *λ*_max_] is the interval of Fourier space used in the disk harmonic expansion, see Ref. (16, Section 2.4).

**Figure 1.**
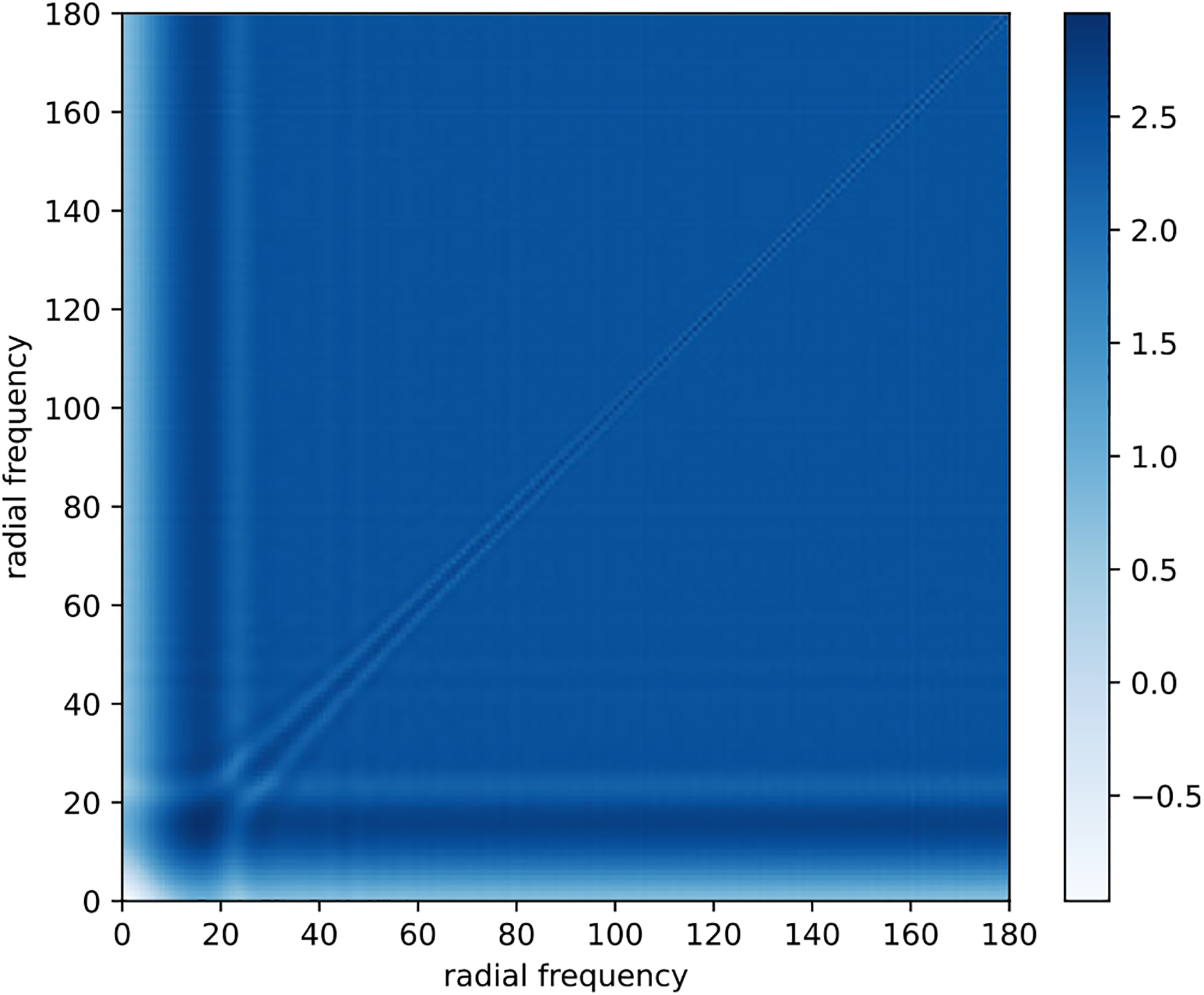
We visualize ${\text{log}}_{10}\left({\sum_{i=1}^{N}{\left|{\hat{h}}_{i}\left(\left|\xi \right|\right)\right|}^{2}\left|{\hat{h}}_{i}\left(\left|\eta \right|\right)\right|}^{2}\right)$ for each pair of radial frequencies ∣ξ∣, ∣η∣ for the experimental dataset EMPIAR-10028^(18)^ obtained from the Electron Microscopy Public Image Archive^(19)^. All values are greater than −1 in the log scale.

Informally speaking, Assumption A3 can be interpreted as saying that for any pair of frequencies *ξ* and *η* there is a point spread function *h*_*i*_ such that neither ${\hat{h}}_{i}(\xi )$ nor ${\hat{h}}_{i}(\eta )$ are zero. Assumption A1 implies that ${\hat{h}}_{i}(\xi )={\hat{h}}_{i}\left(\left|\xi \right|\right)$ is a radial function. Figure 1 shows the values of ${\sum_{i=1}^{N}\left|{\hat{h}}_{i}(\xi )\right|}^{2}{\left|{\hat{h}}_{i}(\eta )\right|}^{2}$ for each pair of radial frequency *ξ*, *η* in log scale, for 1081 distinct CTF images of size *L* = 360, whose defocus values range from 0.81 to 3.87 m, for an experimental dataset; see Section 4 for more details.

This assumption is much weaker than assuming that for all *i* that $\left|{\hat{h}}_{i}\right|$ does not vanish at any frequency. In the latter case, we could just use *h*_*i*_ to invert each equation to get access to the underlying functions *f*_*i*_. If we had direct access to the underlying functions *f*_*i*_, then we could approximate the covariance function *c* by the sample covariance

$$c_{N}\left(x^{\prime },y^{\prime }\right)≔\frac{1}{N-1}\sum_{i=1}^{N}\left[f_{i}\left(x^{\prime }\right)-\tilde{f}\left(x^{\prime }\right)\right]\cdot \left[f_{i}\left(y^{\prime }\right)-\tilde{f}\left(y^{\prime }\right)\right],$$

where $\tilde{f}\left(x^{\prime }\right)=\sum_{i=1}^{N}\frac{1}{N}f_{i}\left(x^{\prime }\right)$ is the sample mean. Indeed, *c*_*N*_(*x*′, *y*′) → c(*x*′, *y*′) by the law of large numbers. To clarify why it is useful for ${\hat{h}}_{i}$ not to vanish, note that in the Fourier domain our measurement model can be expressed as

$${\hat{g}}_{i}={\hat{h}}_{i}{\hat{f}}_{i}+{\hat{\varepsilon }}_{i},$$

where ${\hat{g}}_{i}$, ${\hat{h}}_{i}$, ${\hat{f}}_{i}$, and ${\hat{\varepsilon }}_{i}$ denote the Fourier transforms of *g*_*i*_, *h*_*i*_, *f*_*i*_, and *ε*_*i*_, respectively. Since taking the Fourier transform changes convolution to point-wise multiplication, if each Fourier transform $\left|{\hat{h}}_{i}\right|\geq \delta$ for some *δ* > 0, then we could estimate *c* by first estimating ${\hat{f}}_{i}$ from ${\hat{g}}_{i}$ for *i* = 1, …, *N*, and then using the sample covariance matrix. However, in practice, the CTF ${\hat{h}}_{i}$ is approximately a radial function with many zero-crossings, which means that multiplication by ${\hat{h}}_{i}$ destroys information in the corresponding frequencies, making the restoration from a single image ill-posed.

### 2.3. Fourier–Bessel

The main ingredient in our fast covariance estimation is a fast transform into a convenient and computationally advantageous basis, known as the Fourier–Bessel basis (which consists ofthe harmonics on the disk: eigenfunctions of the Laplacian on the disk that obey Dirichlet boundary conditions). This specific choice of basis has a number of beneficial properties:

- it is orthonormal,
- it is ordered by frequency,
- it is steerable, that is, images can be rotated by applying a diagonal transform to the basis coefficients,
- it is easy to convolve with radial functions, that is, images can be convolved with radial functionsby applying a diagonal transform to the basis coefficients.

These properties have made the Fourier–Bessel basis a natural choice in a number of imaging applications^(7,15,20–22)^ and will be central to the development of our fast covariance estimation method. In polar coordinates (*r*, *θ*) in the unit disk {(*r*, *θ*)*: r* ∈ [0, 1), *θ* ∈ [0, 2π)} the Fourier–Bessel basis functions are defined by

(6) $$\psi_{nk}(r,\theta )=\gamma_{nk}J_{n}\left(\lambda_{nk}r\right)e^{in\theta },$$

where *γ*_*nk*_ is a normalization constant, *J*_n_ is the *n*-th order Bessel function of the first kind (see Ref. 23, Section 10.2), and *λ*_*nk*_ is its *k*th smallest positive root; the indices (*n*, *k*) run over $\mathbb{Z}\times {\mathbb{Z}}_{>0}$.

Recent work^(16)^ has devised a new fast algorithm to expand *L* × *L*-images into ~*L*^2^ Fourier–Bessel basis functions. Informally speaking, given ~*L*^2^ basis coefficients, the algorithm can evaluate the function on an *L* × *L* grid in *O*(*L*^2^log*L*) operations; the adjoint can be computed in the same number of operations, which makes iterative methods fast. Compared to previous expansion methods^(7,22)^, it enjoys both theoretically guaranteed accuracy and lower time complexity.

### 2.4. Key property of Fourier–Bessel basis

A key property of the Fourier–Bessel basis is that convolution with radial functions is diagonal transformations in any truncated basis expansion. More precisely, the following result holds (Ref. 16, Lemma 2.3): suppose that $f=\sum_{(n,k)\in I}\alpha_{nk}\psi_{nk}$ for some index set *I*, and *h* = *h*(|*x*|) is a radial function. Then,

(7) $$P_{ℱ}(f*h)=\sum_{(n,k)\in I}\alpha_{nk}\hat{h}\left(\lambda_{nk}\right)\psi_{nk},$$

where *P*_ℱ_ denotes orthogonal projection onto the span of {*ψ*_*nk*_}_(*n*,*k*) ∈I_, $\hat{h}$ is the Fourier transform of *h*, and *λ*_*nk*_ is the *k*th positive root of *J*_*n*_

We emphasize that the weights of the diagonal transform in Equation (7) are not the coefficients of *h* in the disk harmonic expansion. Indeed, since *h* is radial, it follows from Equation (6) that the coefficients *β*_*nk*_ of *h* in the basis *ψ*_*nk*_ satisfy *β*_*nk*_ = 0 when *n* ≠ 0. Computing the weights $\hat{h}\left(\lambda_{nk}\right)$ from the coefficients *β*_*nk*_ of *h* in the basis, would involve computing weighted sums of the Fourier transforms of the basis functions: $\hat{h}\left(\lambda_{nk}\right)=\sum_{l\in 𝒥}\beta_{0l}{\hat{\psi }}_{0l}\left(\lambda_{nk}\right)$, for some index set 𝒥.

As an alternative to the disk harmonic basis expansion, one can consider simply taking the Fourier transform of (1), which also leads to a diagonal representation of the convolution operator. However, the discrete Fourier transform does not have the steerability property, which is essential for the covariance estimation. Another attempt could be to use the polar Fourier transform. However, this representation is not invariant to arbitrary in-plane rotations, but only to finitely many rotations as determined by the discretization spacing of the grid. These expansions are therefore unsuitable for the goal of this article and we instead use expansions into the Fourier–Bessel basis, although other steerable bases could be considered^(24,25)^. Table 1 summarizes the considerations that make the Fourier–Bessel basis a natural choice of basis.

### 2.5. Block diagonal structure

The steerable property of the Fourier–Bessel basis implies that the 2D-covariance will be block diagonal in this basis. A full representation of an *L*^2^ × *L*^2^ matrix requires *O*(*L*)^4^ elements, but this is reduced to *O*(*L*)^3^ nonzero entries by the block diagonal structure. This block diagonal structure follows from the form of the basis functions and that the distribution of in-plane rotations is assumed to be uniform. Indeed, suppose that $f=\sum_{(n,k)\in I}\alpha_{nk}\psi_{nk}$. For simplicity assume that *f* has mean zero; subtracting the mean will only change the radial components, since the other components corresponding to nonvanishing angular frequencies have zero mean by merely averaging over all possible in-plane rotations. By Assumption A2, the covariance function in polar coordinates satisfies

(8) $$c\left((r,\theta ),\left(r^{\prime },\theta^{\prime }\right)\right)=c\left((r,\theta +\varphi ),\left(r^{\prime },\theta^{\prime }+\varphi \right)\right),$$

for all *φ* in [0, 2π]. The covariance function in Equation (3) can be expanded in a double Fourier–Bessel basis expansion as

(9) $$c\left((r,\theta ),\left(r^{\prime },\theta^{\prime }\right)\right)=\sum_{(n,k)\in I}\sum_{\left(n^{\prime },k^{\prime }\right)\in I}C_{\left(nk,n^{\prime }k^{\prime }\right)}\psi_{nk}(r,\theta )\bar{\psi_{n^{\prime }k^{\prime }}}\left(r^{\prime },\theta^{\prime }\right),$$

where C_(*nk*,*n*′*k*′)_ is the covariance matrix in the Fourier–Bessel basis. Combining Equations (8) and (9) and integrating *φ* over [0, 2π] gives

(10) $$c(r,\theta ),\left(r^{\prime },\theta^{\prime }\right))=\frac{1}{2\pi }\int_{0}^{2\pi }c\left(\left(r,\theta +\varphi ),(r^{\prime },\theta^{\prime }+\varphi \right)\right)d\varphi \;=\sum_{(n,k)\in I}\sum_{\left(n^{\prime },k^{\prime }\right)\in I}C_{\left(nk,n^{\prime }k^{\prime }\right)}\frac{1}{2\pi }\int_{0}^{2\pi }\psi_{nk}(r,\theta +\varphi )\bar{\psi_{n^{\prime }k^{\prime }}}(r^{\prime },\theta^{\prime }+\varphi )d\varphi \;=\sum_{(n,k)\in I}\sum_{\left(n^{\prime },k^{\prime }\right)\in I}C_{\left(nk,n^{\prime }k^{\prime }\right)}\psi_{nk}(r,\theta )\bar{\psi_{n^{\prime }k^{\prime }}}\left(r^{\prime },\theta^{\prime }\right)\frac{1}{2\pi }\int_{0}^{2\pi }e^{i(n-n^{\prime })\varphi }d\varphi \;=\sum_{(n,k)\in I}\sum_{\left(n^{\prime },k^{\prime }\right)\in I}\delta_{n=n^{\prime }}C_{\left(nk,n^{\prime }k^{\prime }\right)}\psi_{nk}(r,\theta )\bar{\psi_{n^{\prime }k^{\prime }}}\left(r^{\prime },\theta^{\prime }\right),$$

where *δ*_*n*=*n*′_ is a Dirac function that is equal to 1if *n* = *n*′ and zero otherwise, and we note that the second to last equality uses the fact that *ψ*_*nk*_(*r*, *θ*) = *γ*_*nk*_*J*_*n*_(*λ*_*nk*_*r*)*e*^*inθ*^. Since the coefficients *C*_(*nk*,*n*′*k*′)_ in the expansion Equation (9) are unique, it follows from Equation (10) that C_(*nk*,*n*′*k*′)_ = 0 when *n* ≠ *n*′. Hence, the covariance matrix C_(*nk*,*n*′*k*′)_ has a block diagonal structure whose blocks consist of the indices (*nk*, *nk*′) for a given value of *n*. In the following section, we show how these properties enable a fast method to estimate the covariance matrix.

### 2.6. Covariance estimation

In the Fourier–Bessel basis, Equation (1) is written as

(11) $$G_{i}=H_{i}⊙F_{i}+E_{i},$$

where *G*_*i*_, *F*_*i*_, and *E*_*i*_ are coefficient vectors of *g*_*i*_, *f*_*i*_, *ε*_*i*_ in the Fourier–Bessel basis, respectively and *H*_*i*_ is the vector encoding the convolution operator of Section 2.4, that is, with components $\hat{h_{i}}\left(\lambda_{nk}\right)$. The vectors are *b*-dimensional column vectors, where *b* = *O*(*L*^2^) is the number of basis coefficients. We use this simple structure to obtain a closed-form expression for the sample covariance matrix of the *F*_*i*_. We estimate this matrix by minimizing the discrepancy between the sample covariance and the population covariance; more precisely, the estimated covariance matrix $\tilde{C}$ is computed by solving the least squares-problem

(12) $$\tilde{C}=\underset{c}{\text{arg min}}{\sum_{i=1}^{N}\left\|\left(G_{i}-H_{i}⊙\tilde{\mu }\right){\left(G_{i}-H_{i}⊙\tilde{\mu }\right)}^{T}-\left(C⊙\left(H_{i}H_{i}^{T}\right)+\sigma^{2}I\right)\right\|}_{F}^{2}.$$

where $\tilde{\mu }$ is defined by

(13) $$\tilde{\mu }=\underset{c}{\text{arg min}}{\sum_{i=1}^{N}\left\|G_{i}-H_{i}⊙\mu \right\|}^{2},$$

whose solution is

(14) $$\tilde{\mu }=\left(\sum_{i=1}^{N}H_{i}⊙G_{i}\right)⊘\left(\sum_{i=1}^{N}H_{1}^{⊙2}\right).$$

The least squares-solution of Equation (12) can be determined by the following system of linear equations

(15) $$\left(\sum_{i=1}^{N}H_{i}^{⊙2}{\left(H_{i}^{⊙2}\right)}^{T}\right)⊙\tilde{C}=\sum_{i=1}^{N}\left(H_{i}H_{i}^{T}\right)⊙B_{i}-\sigma^{2}\sum_{i=1}^{N}\text{diag}\left(H_{i}^{⊙2}\right),$$

where

(16) $$B_{i}=\left(G_{i}-H_{i}⊙\tilde{\mu }\right){\left(G_{i}-H_{i}⊙\tilde{\mu }\right)}^{T}.$$

It follows that

(17) $$\tilde{C}=\left(\sum_{i=1}^{N}\left[B_{i}⊙\left(H_{i}H_{i}^{T}\right)-\sigma^{2}\text{diag}\left(H_{i}^{⊙2}\right)\right]\right)⊘\left(\sum_{i=1}^{N}H_{i}^{⊙2}{\left(H_{i}^{⊙2}\right)}^{T}\right).$$

As discussed in Section 2.5, the covariance matrix is block diagonal in the Fourier–Bessel basis. More precisely, the only nonzero elements $\tilde{C}\left(nk,n\prime k\prime \right)$ of the matrix $\tilde{C}$ are those with *n*= *n*′. Therefore, the matrices in Equations (16) and (17) need only be calculated for this subset of indices. Since there is a total of *O*(*L*^3^) of these indices, this reduces the computational complexity compared to computing with the full matrices.

Note that the covariance matrix estimated from (Equation 17) may not be positive semidefinite due to subtraction of the term $\sigma^{2}\text{diag}\left(H_{i}^{⊙2}\right)$. Therefore, when running the method in practice it is beneficial to use an eigenvalue shrinkage method. The computational cost of eigenvalue shrinkage for a matrix with our block structure is *O*(*L*^4^) ^(15,26)^. For completeness, we include this computational cost of eigenvalue shrinkage in our overall computational complexity. Informally speaking, the idea of eigenvalue shrinkage is to replace the term $\sum_{i=1}^{N}\left[B_{i}⊙\left(H_{i}H_{i}^{T}\right)-\sigma^{2}\text{diag}\left(H_{i}^{⊙2}\right)\right]$ in Equation (17) by $\sum_{i=1}^{N}\left[B_{i}⊙\left(H_{i}H_{i}^{T}\right)\right]$, and then shrink and truncate the eigenvalues in a systematic way before Hadamard division^(26)^. The steps of the algorithm are summarized in Algorithm 1.


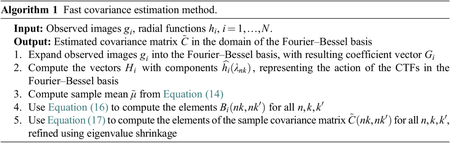


The complexity of step 1 of the algorithm is *O*(*NL*^2^log*L*). The complexity of step 2 is *O*(*ML*^2^log*L*). The complexity of step 3 is *O*(*NL*^2^), since the number of basis coefficients is *O*(*L*^2^). The complexity of step 4 is *O*(*NL*^3^), since there are at most *O*(*L*^2^) nonzero elements with indices (*n*_*k*_, *n*_*k*_′). The complexity of step 5 is *O*(*NL*^3^ + *L*^4^), where the additional term 𝒪(*L*^4^) comes from the computational complexity of eigenvalue shrinkage. Thus, the total complexity of the algorithm is *O* (*NL*^3^ + *L*^4^).

We now show how this can be used in an application to image denoising. Given an estimate of the covariance matrix $\tilde{C}$, the CWF approach estimates the *F*_*i*_ by a linear Wiener filter^(15)^,

(18) $${\tilde{F}}_{i}=\tilde{\mu }+\tilde{C}\text{diag}\left(H_{i}\right){\left(\tilde{C}⊙\left(H_{i}H_{i}^{T}\right)+\sigma^{2}I\right)}^{-1}\left(G_{i}-H_{i}⊙\tilde{\mu }\right),$$

See Ref. 15 for more details.

## 3. Synthetic Data Results

We compare the timings of our fast method to previous approaches^(15)^, for synthetic images generated from the 3-D volume of SARS-CoV-2 (Omicron) spike complexes^(27)^ (EMD-32743), from the online EM data bank^(28)^. The original volume has size 512in each dimension, with pixel size 0.832 Å. We downsample the original volume to size *L* × *L* × *L*, with *L* = 32,128 and 512, respectively, and show the computational times. To generate the synthetic noisy images, we first generate 10,000 clean projection images of the 3-D volume fromrandom and uniformly distributed viewing directions. We next divide the set of clean images into a number of defocus groups, where the defocus values range from1 *μ*m to 4 *μ*m. For all CTFs, we set the voltage as 300 kV and the spherical aberration as 2 mm. After convolving the images with their CTFs, we add colored noise with power spectral density 1/(*rL*/20+1) up to a constant scale, where *r* ∈[0, 1] is the radial frequency. For both the previous method and ours, the CTFs and the noisy images are whitened before estimating the covariance. A few sample clean and noisy images are shown in Figure 5. All experiments were carried out on a machine with 750 GB memory and 72 Intel Xeon E7–8880 v3 CPUs running at 2.30GHz.We note that our implementation heavily relies on packages such as FINUFFT^(29,30)^, NumPy^(31)^, and SciPy^(32)^, which are not fully optimized for parallel computing. The implementation of our fast method and the code of CWF^(15)^ both effectively use only around 20 cores at runtime. Due to the complexity of modern computational architectures, performing a fair comparison can be challenging, but we have made every effort to do so. Both our code and the CWF code^(15)^ take input images in a tensor format, and used vectorized operations whenever possible to avoid for-loops. The primary factor determining performance is not the specific implementation of the code, but rather the underlying computational complexities. Improving the parallelization is a technical direction for future work.

**Figure 2.**
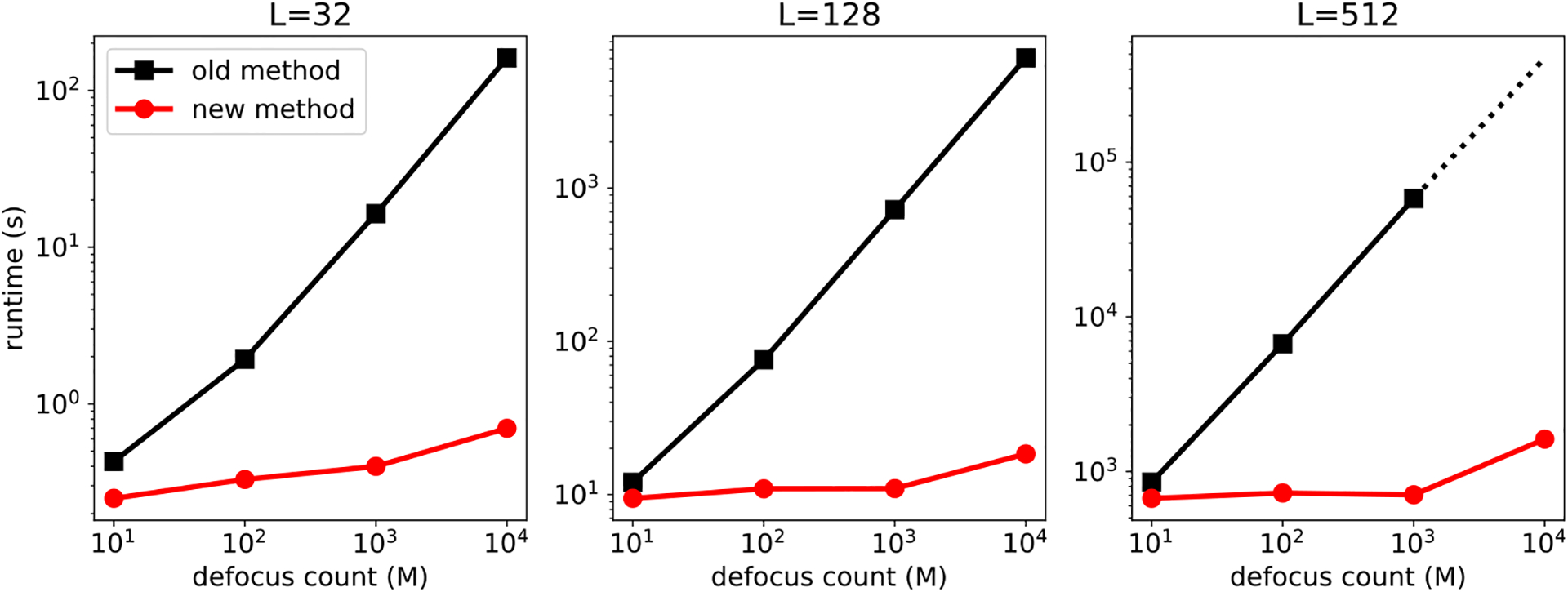
Timing comparison for covariance matrix estimation of 10,000 images of size L × L. The old method^(15)^ timing for L= 512 and defocus count 10^4^ is extrapolated due to time and memory constraints.

Figure 2 shows the time required to estimate the covariance matrices as a function of the number of defocus groups. Note that the runtime for the previous approach for the largest image and defocus group sizes is infeasibly large and that our fast method exhibits a speedup of up to three orders of magnitude.

**Figure 3.**
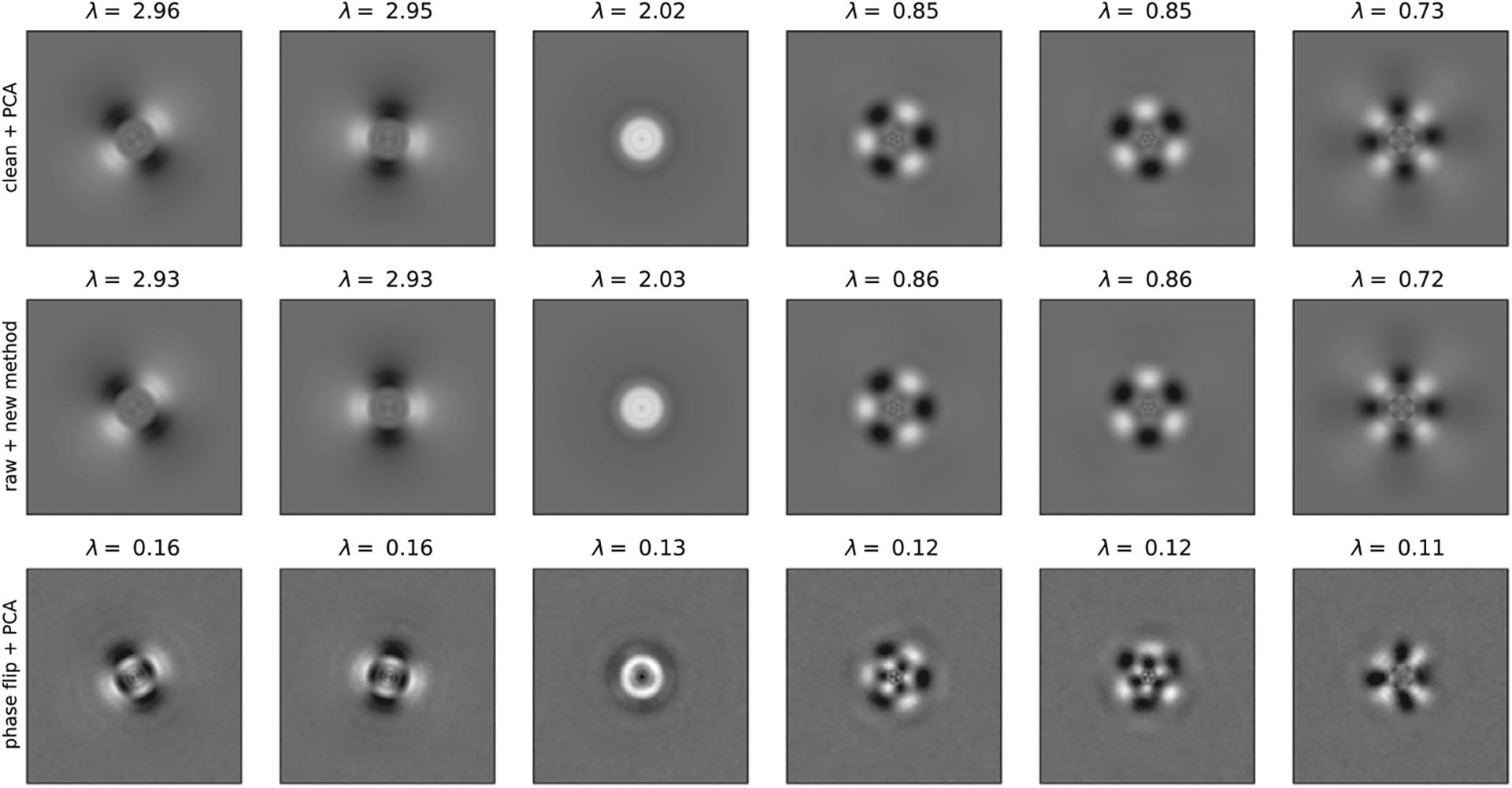
Top six eigenimages computed by traditional PCA on 10^6^ clean images (top panel), our new method on 10^4^ raw images (middle panel), and traditional PCA on 10^4^ phase-flipped images (bottom panel). The signal-to-noise ratio for the images for the new method and traditional PCA was 0.1.

Figure 3 shows the top six principal components estimated by our method and by traditional PCA using 10^4^ raw images, where *L*=128, SNR=0.1 and *M* =100 defocus groups, compared to traditional PCA on 10^6^ images. We use the sample covariance matrix of phase-flipped images in real space for the traditional PCA. For all methods, we use *λ* to denote 100 times the eigenvalues of the eigenimages. The eigenimages from the traditional PCA look much noisier than ours, and contain artifacts that are due to imperfect CTF correction (see, e.g., the circular artifacts in top three eigenimages in Figure 3). The eigenimages from the traditional PCA also fail to preserve the symmetries (see, e.g., 6th eigenimage in Figure 3) that are present in our eigenimages, since they do not utilize the steerable basis and rotation-augmented images.

**Figure 4.**
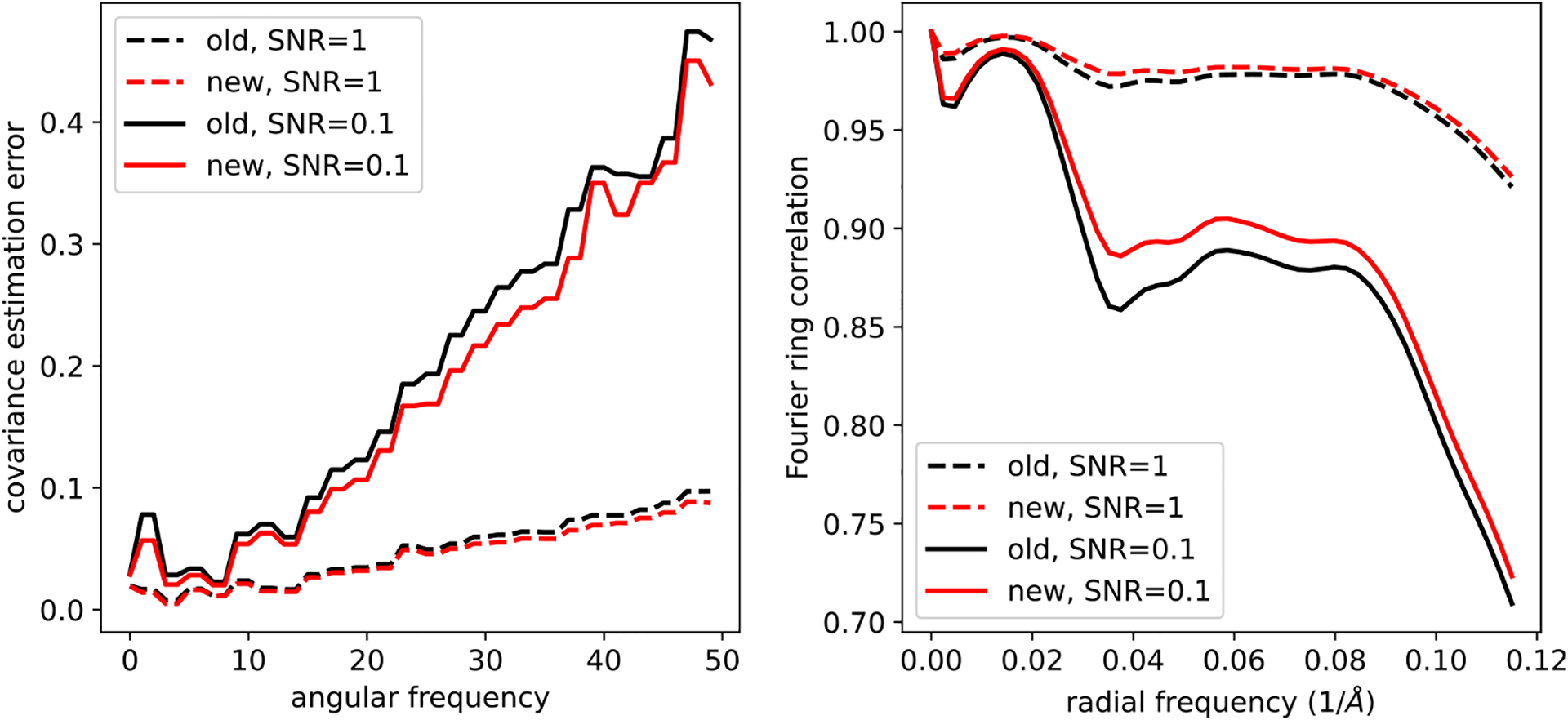
Relative estimation error of the covariance matrix (left) and the Fourier ring correlation between the denoised and clean images (right).

Figure 4 shows the quality of covariance estimation and image denoising when *L*= 128 and using *M* =100defocus groups. The quality of the covariance estimation is measured by the relative error in each angular frequency, which is defined as

(19) $${\text{err}}_{n}={\left\|C_{n}-{\tilde{C}}_{n}\right\|}_{F}/{\left\|C_{n}\right\|}_{F},$$

where *C*_*n*_ and ${\tilde{C}}_{n}$ are respectively the *n*th diagonal blocks of the clean and estimated covariance matrix, corresponding to all indices of the form (*n*_*k*_, *n*_*k*_′). The clean covariance matrix was approximated by the sample covariance matrix of 10^6^ clean projection images. The performance of image denoising is measured by the Fourier ring correlation (FRC) between the clean and denoised images. Namely, for the ith pair of clean and denoised images $I_{i}^{c}$ and $I_{i}^{d}$, we first compute their Fourier coefficient vectors $f_{i,r}^{c}$, $f_{i,r}^{d}$ at radial frequency *r* by the nonuniform FFT^(33–35)^, where 1 ≤ *i* ≤ *N* and 0 ≤ *r* ≤ *r*_max_. We then compute their averaged correlation for each *r*:

$$\text{FRC}(r)=\frac{1}{N}\sum_{i=1}^{N}\frac{\langle f_{i,r}^{c},f_{i,r}^{d}\rangle }{\left\|f_{i,r}^{c}\right\|\left\|f_{i,r}^{d}\right\|},$$

where 〈·, ·〉denotes the inner product between two complex vectors. The FRC is a real-valued quantity due to a symmetry property that arises since the images $I_{i}^{c}$, $I_{i}^{d}$ are real-valued.

**Figure 5.**
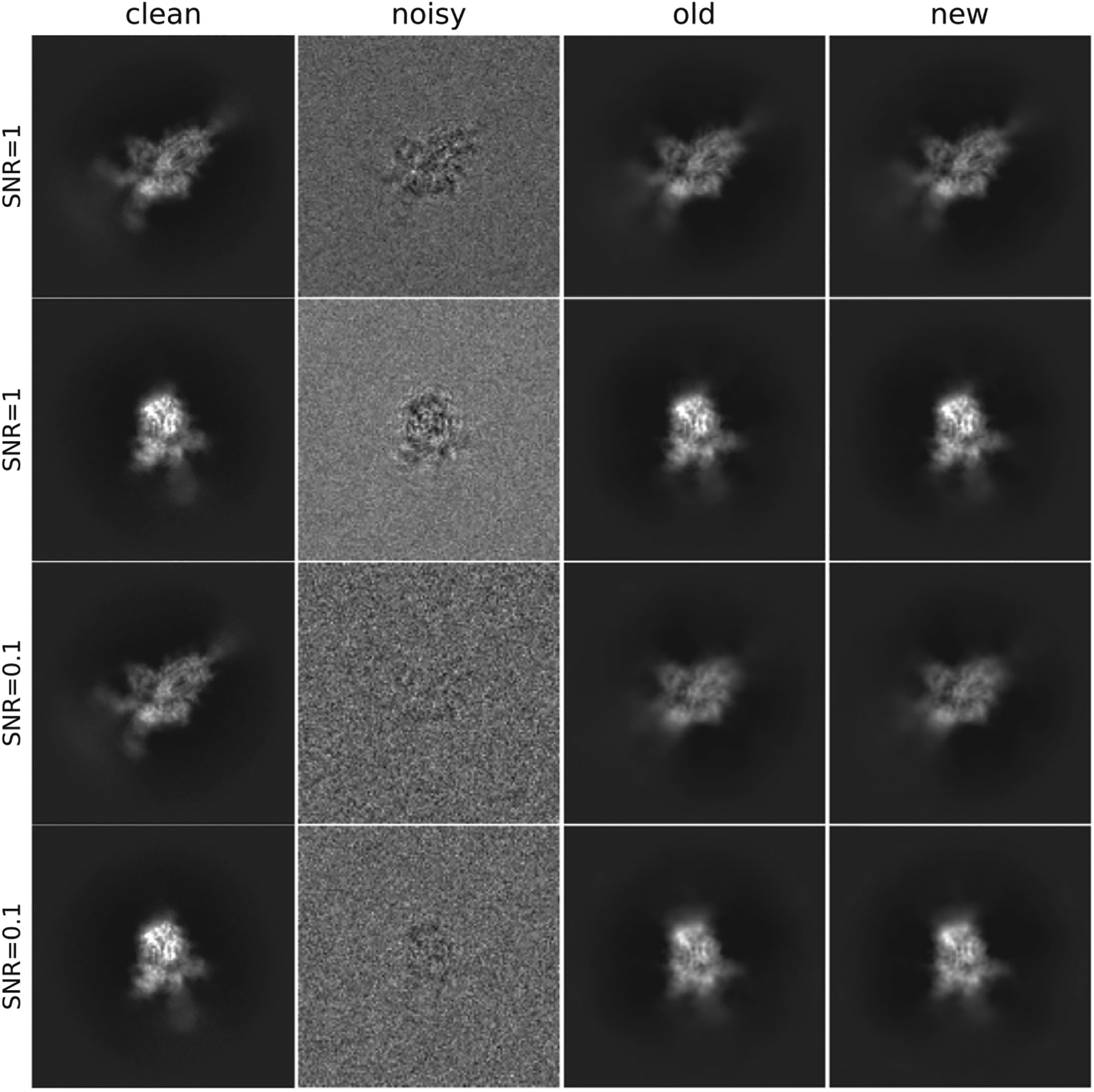
Clean, noisy and denoised images. The covariance estimation used N = 10,000 images, and parameters L= 128 and M = 100.

In addition to the speedups of Figure 2, the left panel of Figure 4 demonstrates a slight increase in the estimation quality of our proposed algorithm, compared to the previous approach. This is possibly caused by improved accuracy in the Fourier–Bessel basis approximation as well as improved accuracy by using the closed-form expression Equation (17) compared to the approximate conjugate gradient step of previous approaches. Similarly, on the right panel, the Fourier ring correlation between the denoised and clean images shows a slight performance increase. Figure 5 shows sample denoised images for different values of the SNR where *L*= 128 and *M* = 100. As a comparison, we show images denoised using the approach of this paper and the CWF method^(15)^.

## 4. Experimental Data Results

We conclude by using our method on two experimental datasets obtained from the Electron Microscopy Public Image Archive^(19)^, namely EMPIAR-10028^(18)^ and EMPIAR-10081^(36)^. EMPIAR-10028 is a dataset of the Plasmodium falciparum 80S ribosome bound to the antiprotozoan drug emetine whose 3-D reconstruction is available in the EM data bank as EMD-2660^(18)^. The dataset contains 105247 motion corrected and picked particle images, from 1081 defocus groups, of size 360 × 60 with 1.34 Å pixel size. EMPIAR-10081 is a dataset of the human HCN1 hyperpolarization-activated cyclic nucleotide-gated ion channel, whose 3-D reconstruction can be found in the EM data bank as EMD-8511^(36)^. The dataset contains 55,870 motion corrected and picked particle images, from 53,384 defocus groups, of size 256 × 256 with 1.3 Å pixel size.

Computational times are shown in Table 2, showing a speedup of more than two orders of magnitude for the datasets with the largest number of distinct CTFs. Note that regular CWF on EMPIAR-10081 encounters memory issues and cannot be run to completion, whereas our fast method runs seamlessly. The old CWF has much higher space complexity *O*(*ML*^3^) for covariance estimation than that of our method *O*(*L*^3^). The reason is that the old CWF represents CTFs using block diagonal matrices (each takes *O*(*L*^3^) memory). Moreover, the old CWF loads all these block diagonal CTFs at once in memory so as to define the linear operator for the conjugate gradient method. Our method circumvents the need to store all CTFs at once due to our closed-form solution, and therefore we can read CTFs in batches. Moreover, each CTF has diagonal representation in our method, which means much lower space complexity *O*(*L*^2^). The *O*(*L*^3^) space complexity for our method is only for storing a single covariance matrix.

For EMPIAR-10081, storing the CTFs for the old method takes approximately 50,000 × 256^3^/6) × 8/10^9^ ≈1118 GB which is above the 750 GB memory limit of our machine. For the new method, storing the covariance matrix takes 256^3^/6) × 8/10^9^ ≈0.02 GB and storing the CTFs takes 1000 × 256^2^ × 8/10^9^ ≈0.52 GB (with batch size 1000) that can be even handled by a common laptop.

**Figure 6.**
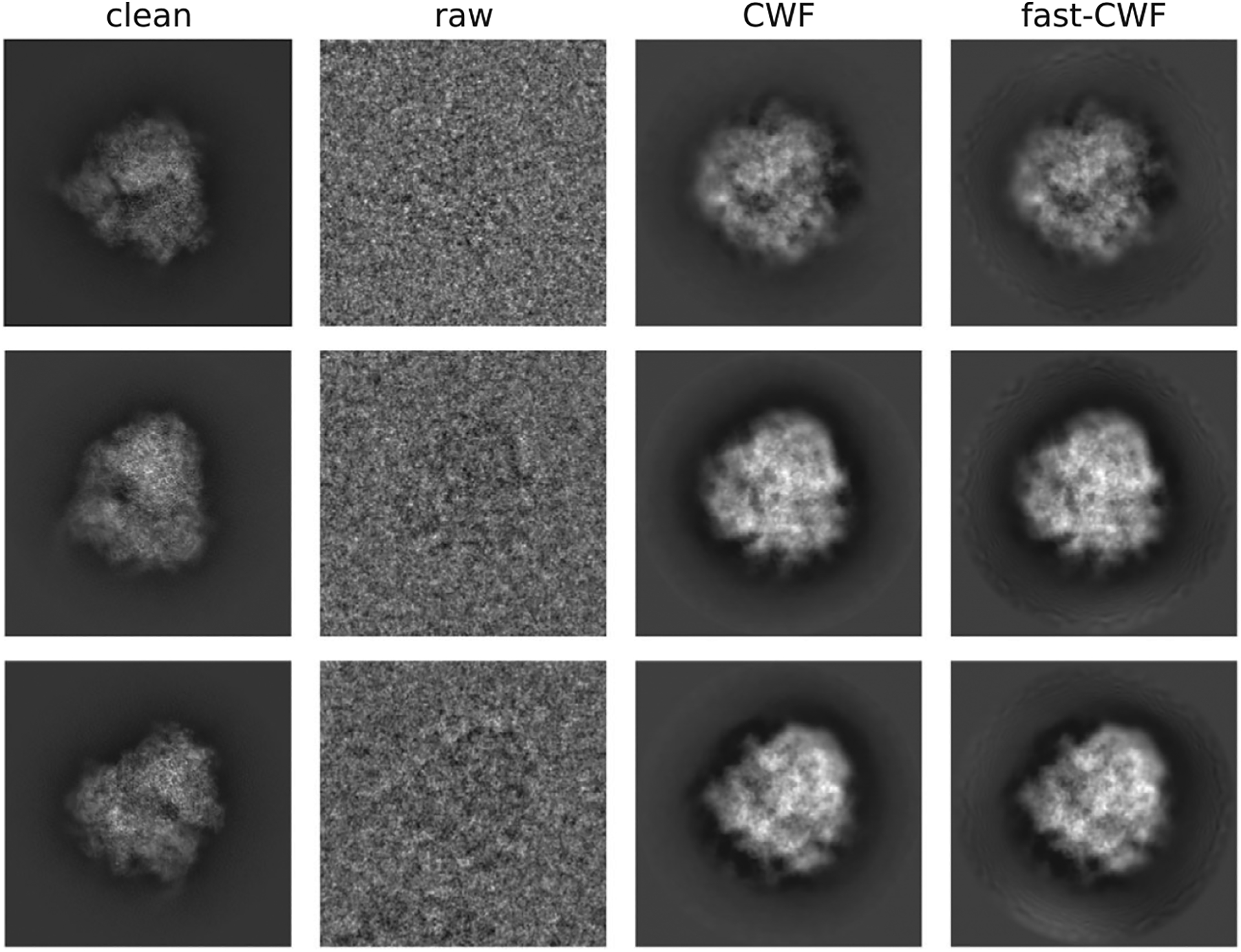
Denoised images (EMPIAR-10028). The method used N = 105,247 images, from M = 1081 defocus groups, of size 360 × 360. The clean images are obtained by aligning 1000 clean projection images (from uniformly distributed viewing directions) with phase-flipped raw images.

**Figure 7.**
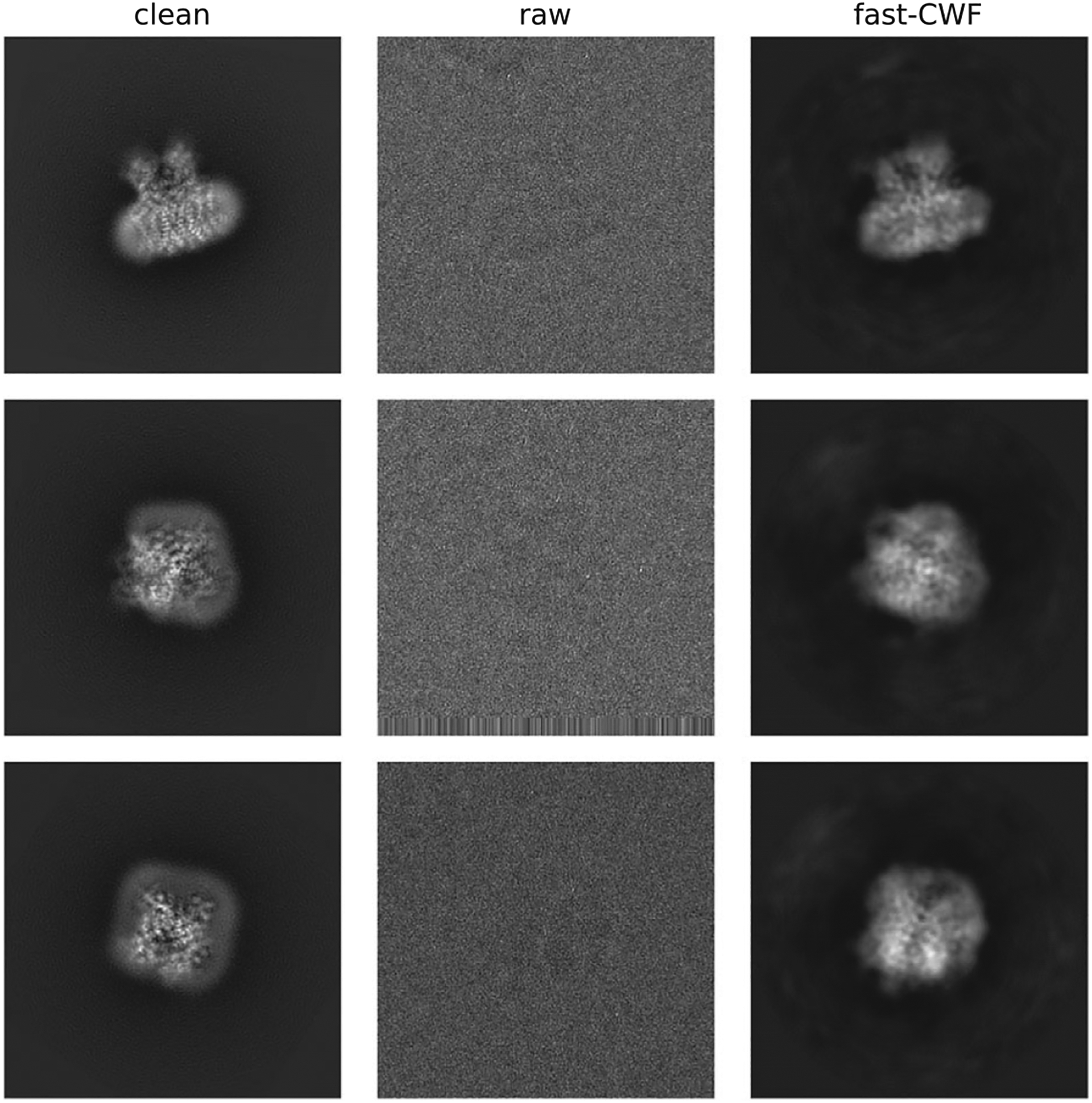
Denoised images (EMPIAR-10081). The method used N = 55,870 images, from M = 53,384 defocus groups, of size 256 × 256. The clean images are obtained by aligning 1000 clean projection images (from uniformly distributed viewing directions) with phase-flipped raw images.

In order to obtain a comparison, we therefore additionally downsample these images to *L*= 128 where the original CWF can successfully run. On EMPIAR-10028, we used all images for covariance estimation, and denoised the 2014 images from 0 th, 50 th, 100 th, …, 1000 th defocus groups. On EMPIAR-10081, we used all images for covariance estimation, and denoised the 502 images from 0 th, 100 th, 200 th, …, 50,000 th defocus groups. For both old and new methods, the covariance matrices are further refined to correct image contrast variations^(21)^.Sample visualization results are shown in Figures 6 and 7. The old CWF method cannot handle the full resolution recovery for EMPIAR-10081 (with about 50,000 distinct CTFs) due to its high space complexity. Moreover, the old CWF method does not return satisfying restored images for downsampled low resolution images. The reason is that for EMPIAR-10081, almost all images belong to different defocus groups, and the estimated noise power spectrum from a single image is too noisy. Therefore, EMPIAR-10081 is a failure case for the old CWF method in terms of both accuracy and computation time. In contrast, our method assumes radially symmetric noise power spectrum and estimates it using NUFFT over concentric rings with different radii, and averages over each ring. This averaging process largely reduces the noise in the estimated power spectrum.

## 5. Discussion

Covariance estimation and PCA of cryo-EM images are key ingredients in many classic cryo-EM methods including multivariate statistical analysis^(3–6)^ and Kam’s method for ab initio modeling^(8)^. We propose a fast method to estimate the covariance matrix of noisy cryo-EM images, and then illustrate its application to simultaneously correct for the CTFs and denoise the images. The approach relies on recent improvements to algorithms for expanding images in the Fourier–Bessel basis^(16)^, and has time complexity *O*(*NL*^3^+*L*^4^) which is independent of the number of defocus groups. Our new approach is both significantly faster and more memory-efficient compared to the previous CWF method^(15)^ and we apply our method to large experimental datasets with many distinct CTFs with speedups by factors up to more than two orders of magnitude. Our approach could potentially be extended to higher-dimensional data and to the setting where images are distorted by CTFs which are not exactly radial, using either analytical correction terms or iterative numerical steps.

## Figures and Tables

**Table 1.** Summary of desirable properties of a few different basis candidates.

Basis	Orth.	Cont. steerable	Radial convolution diag.	Fast expansion^a^
Real	✓	✗	✗	✓
2-D discrete Fourier	✓	✗	✓	✓
Polar Fourier	✗	✗ (discrete)	✓	✓
PSWF^(24,25)^	✓	✓	✗	✗
Fourier-Bessel^(16)b^	✓	✓	✓	✓

a The basis expansion from Cartesian grid representation can be completed within *O*(*L*^2^) operations up to log factors.

b The new expansion algorithm^(16)^ improves the previous computational method^(7)^ in terms of accuracy guarantees, computational complexity, and the fact that it derives weights such that radial convolution is a diagonal operation.

**Table 2.** Timing comparison in seconds for EMPIAR-10028 (top) and EMPIAR-10081 (bottom).

EMPIAR-10028					
Methods	*T*_ffb_	*T*_ctf_	*T*_cov_	*T*_denoise_	*T*_total_
Old CWF, *L* = 360	1415	598	27550	201	29764
Fast CWF, *L* = 360	768	5	2220	95	3088
EMPIAR-10081					
Methods	*T*_ffb_	*T*_ctf_	*T*_cov_	*T*_denoise_	*T*_total_
Old CWF, *L* = 128	47	3369	46318	45	49779
Fast CWF, *L* = 128	35	8	93	27	163
Old CWF, *L* = 256	363	NA	NA	NA	NA
Fast CWF, *L* = 256	169	34	1007	163	1373

*Note*. For EMPIAR-10081, when *L*=256, the old CWF method^(15)^ encounters memory issues and cannot be run until completion. *T*_ffb_, the time required to expand all images in the Fourier–Bessel basis (step 1 in Algorithm 1); *T*_ctf_, the time to compute amatrix representation of the application of the point spread function (step 2 in Algorithm 1); *T*_cov_, the time to estimate the covariance matrix (steps 3–5 in Algorithm 1); *T*_denoise_, the time to denoise the number of images indicated in the main text (2014 images for EMPIAR-10028 and 502 images for EMPIAR-10081); *T*_total_, the total computational time.

## Data Availability

Replication data and code can be found at https://github.com/yunpeng-shi/fast-cryoEM-PCA.

## Impact Statement

Singe particle reconstruction in cryo-electron microscopy (cryo-EM) is an increasingly popular technique for near-atomic imaging of biological macromolecules. Both technological and computational advances have driven the progress of the techniques, yet many computational obstacles still remain. We introduce a fast method to estimate the covariance matrix of noisy cryo-EM images, which is a central component of many computational cryo-EM techniques. As an application, we use the covariance matrix for image denoising and deconvolution of the microscope’s contrast transfer function. Our method provides orders of magnitude speedup compared to previous approaches, which opens the door to tackling more challenging datasets.

## Footnotes

1 Code is available at https://github.com/yunpeng-shi/fast-cryoEM-PC.A.
